# 3,5,5,6,8,8-Hexamethyl-5,6,7,8-tetra­hydro-2-naphthoic acid (AHTN–COOH)

**DOI:** 10.1107/S1600536810038572

**Published:** 2010-09-30

**Authors:** Paul Kuhlich, Robert Göstl, Ramona Metzinger, Christian Piechotta, Irene Nehls

**Affiliations:** a BAM Federal Institute for Materials Research and Testing, Richard-Willstätter-Strasse 11, D-12489 Berlin, Germany; b Humboldt-Universität zu Berlin, Department of Chemistry, Brook-Taylor-Strasse 2, D-12489 Berlin, Germany

## Abstract

The title compound, C_17_H_24_O_2_, is the product of a haloform reaction of 6-acetyl-1,1,2,4,4,7-hexa­methyl­tetra­line (AHTN). The compound is a racemic mixture with a disorder in its aliphatic ring [occupany ratio 0.683 (4):0.317 (4)] due to two possible half-chair forms. The carb­oxy­lic acid unit is slightly twisted out of coplanarity with the aromatic system [dihedral angle = 29.26 (6)°]. In the crystal, pairs of short classical inter­molecular O—H⋯O hydrogen bonds link pairs of mol­ecules around a center of symmetry.

## Related literature

For a similar synthesis of AHTN-COOH and the mechanism of the haloform reaction, see: Valdersnes *et al.* (2006 ▶); Fuson & Bull (1934 ▶). For the crystal structure of AHTN, see: De Ridder *et al.* (1990 ▶). For environmental occurrence and estrogenic activity of AHTN, see: Heberer (2003 ▶); Bitsch *et al.* (2002 ▶). For industrial synthesis of AHTN and annual production amounts, see: Sell (2006 ▶); Kupper *et al.* (2004 ▶).
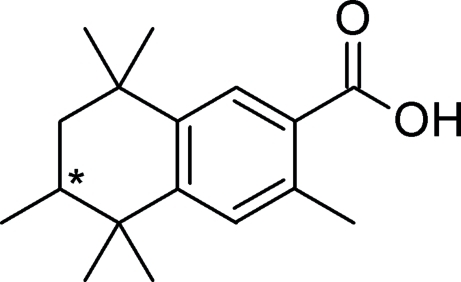


## Experimental

### 

#### Crystal data

C_17_H_24_O_2_
*M* *_r_* = 260.36
Monoclinic, 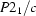

*a* = 8.9718 (2) Å
*b* = 10.1447 (3) Å
*c* = 17.7058 (5) Å
β = 112.3100 (19)°
*V* = 1490.88 (7) Å^3^
*Z* = 4
Mo *K*α radiation
μ = 0.07 mm^−1^
*T* = 100 K
0.44 × 0.44 × 0.28 mm

#### Data collection

Stoe IPDS-2t diffractometer
5695 measured reflections
2933 independent reflections
2525 reflections with *I* > 2σ(*I*)
*R*_int_ = 0.016

#### Refinement

*R*[*F* ^2^ > 2σ(*F* ^2^)] = 0.047
*wR*(*F* ^2^) = 0.130
*S* = 1.04
2933 reflections
206 parameters
30 restraints
H-atom parameters constrained
Δρ_max_ = 0.29 e Å^−3^
Δρ_min_ = −0.34 e Å^−3^

### 

Data collection: *X-AREA* (Stoe & Cie, 2002 ▶); cell refinement: *X-AREA*; data reduction: *X-RED32* (Stoe & Cie, 2002 ▶); program(s) used to solve structure: *SHELXS97* (Sheldrick, 2008 ▶); program(s) used to refine structure: *SHELXL97* (Sheldrick, 2008 ▶); molecular graphics: *PLATON* (Spek, 2009 ▶); software used to prepare material for publication: *PLATON*.

## Supplementary Material

Crystal structure: contains datablocks global, I. DOI: 10.1107/S1600536810038572/fl2316sup1.cif

Structure factors: contains datablocks I. DOI: 10.1107/S1600536810038572/fl2316Isup2.hkl

Additional supplementary materials:  crystallographic information; 3D view; checkCIF report

## Figures and Tables

**Table 1:** Hydrogen-bond geometry (Å, °)

*D*—H⋯*A*	*D*—H	H⋯*A*	*D*⋯*A*	*D*—H⋯*A*
O2—H2⋯O1^i^	0.91	1.72	2.6305 (16)	178

Symmetry code: (i) 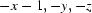.

**Fig. 1.**
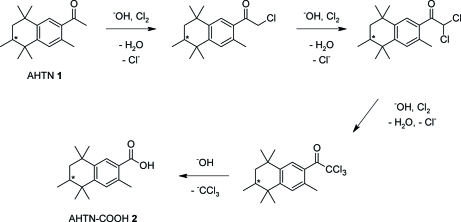
Haloform reaction of AHTN 1 to result in AHTN-COOH 2.

**Fig. 2.**
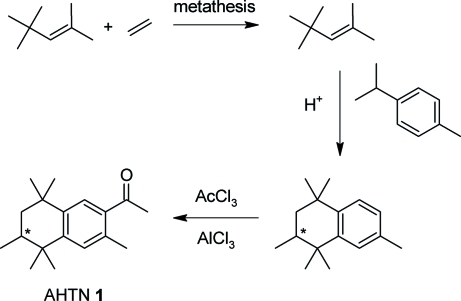
Industrial production of AHTN 1.

**Fig. 3.**
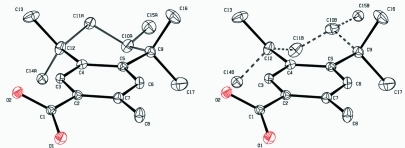
Displacement parameter plots of the two half-chair conformations in the crystal structure. Displacement ellipsoids are set to 30% probability, H atoms were excluded for clarity.

**Fig. 4.**
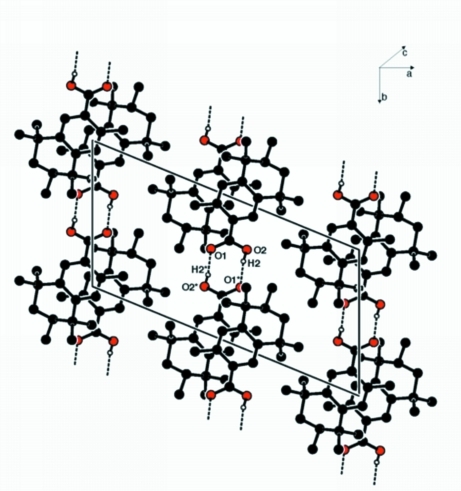
Packing diagram of the unit-cell content, view direction [0 1 0]. All H atoms are excluded except for the one involved in H bond formation. Only one conformer is shown.

## supplementary crystallographic information

### Comment

The title compound is the carboxylic acid AHTN-COOH 2 of 6-acetyl-1,1,2,4,4,7-hexamethyltetraline (AHTN) obtained by a Haloform reaction (Fuson & Bull, 1934) of AHTN 1 with sodium hypochlorite solution (NaOCl). The reaction mechanism is shown in Figure 1. A similar synthesis was given by Valdersnes *et al.* (2006). AHTN-COOH 2 might be a disinfection by-product of AHTN 1.

AHTN itself is a widely used fragrance in cosmetics and cleaning products. Its commonest trade name is Tonalide. The crystal structure of AHTN was shown by De Ridder (De Ridder *et al.*, 1990). AHTN is produced in the ton-scale, in 2000, approx. 343,000 kg (Kupper *et al.*, 2004) and introduced into the environment mainly by sewage treatment plants supplied by municipal wastewater. It can be found in surface water at low µg/L concentration (Heberer, 2003). Due to the low estrogenic potential of AHTN (Bitsch *et al.*, 2002) this might induce a health concern.

The industrial synthesis of AHTN 1 is shown in Figure 2. Both starting chemicals are inexpensive and readily available: *para*-cymene and *neo*-hexene, the later results from an olefin metathesis of di-*iso*-butylene with ethylene (Sell, 2006). AHTN 1 is obtained as racemic mixture in industrial-scale. Hence, the title compound AHTN-COOH 2 is obtained as racemic mixture, too.

The compound (Fig. 3) is crystallizing in the monoclinic space group P21/c. It shows disorder (68:32) within the cyclohexane moiety: the two possible half-chair forms are present.

The compound exhibits a short classical intermolecular H bond which links the molecules into pairs around a center of symmetry (Fig. 4). A summary of these interactions is compiled in Table 1.

### Experimental

NaOCl was added to a solution of racemic AHTN in acetonitrile. The mixture was stirred for 72 h at room temperature. Afterwards water was added to dissolve precipitated salt, sodium sulfite to quench free chlorine and 6 M hydrochloric acid to adjust the pH to one. The organic layer was extracted with diethyl ether. The extracts were combined, dried over anhydrous sodium sulfate and filtered. Evaporation of the solvent *in vacuo* gave a white crystalline residue that was washed with cyclohexane. Recrystallization from diethyl ether resulted in colorless crystals [m.p. 224°C]. IR (ν, cm^-1^): 1675(s), 1609(s), 1550(s), 1498(s), 1364(s), 1305(s), 1262(s), 1247(s), 1111(s), 912(s), 885(s), 850(s); ^1^H-NMR (500 MHz, CD_3_OD, TMS): δ [ppm] = 7.88 (1H, s), 7.24 (1H, s), 2.52 (3H, s), 1.89 (1H, m), 1.64 (1H, t), 1.41 (1H, dd), 1.33 (3H, s), 1.30 (3H, s), 1.25 (3H, s), 1.07 (3H, s), 1.01 (3H, d); ^13^C-NMR (125 MHz, CD_3_OD, TMS): δ [ppm] = 171.5, 151.6, 143.4, 137.8, 131.3, 130.5, 128.5, 44.7, 38.9, 35.8, 35.0, 32.8, 32.4, 28.9, 25.1, 21.9, 17.3; (+)-ESI/MS: 261.5 (40) [M+H^+^], 283.5 (100) [M+Na^+^].

### Refinement

Non-H atoms were refined anisotropically and all H atoms were placed in calculated positions and refined using a riding model with C—H distances of 0.93 Å for the CH groups, 0.97 Å for the CH_2_ groups and 0.96 Å for the CH_3_ groups, *U*_iso_(H) = 1.5*U*_eq_(non-H), except for the H2 atom which was found in the electron density map and fixed in its position. Methyl groups were allowed to rotate as rigid groups.

### Crystal data

**Table:**

C_17_H_24_O_2_	*F*(000) = 568
*M**_r_* = 260.36	*D*_x_ = 1.160 Mg m^−^^3^
Monoclinic, *P*2_1_/*c*	Mo *K*α radiation, λ = 0.71073 Å
Hall symbol: -P 2ybc	Cell parameters from 30064 reflections
*a* = 8.9718 (2) Å	θ = 2.3–30.9°
*b* = 10.1447 (3) Å	µ = 0.07 mm^−^^1^
*c* = 17.7058 (5) Å	*T* = 100 K
β = 112.3100 (19)°	Fragment, colourless
*V* = 1490.88 (7) Å^3^	0.44 × 0.44 × 0.28 mm
*Z* = 4	

### Data collection

**Table:**

Stoe IPDS-2t diffractometer	2525 reflections with *I* > 2σ(*I*)
Radiation source: long fine focus sealed X-ray tube	*R*_int_ = 0.016
planar graphite	θ_max_ = 26.0°, θ_min_ = 2.8°
Detector resolution: 6.67 pixels mm^-1^	*h* = −11→10
ω–rotation,ω–incr.=1°,319 exposures scans	*k* = −12→12
5695 measured reflections	*l* = −21→10
2933 independent reflections	

### Refinement

**Table:**

Refinement on *F*^2^	Primary atom site location: structure-invariant direct methods
Least-squares matrix: full	Secondary atom site location: difference Fourier map
*R*[*F*^2^ > 2σ(*F*^2^)] = 0.047	Hydrogen site location: inferred from neighbouring sites
*wR*(*F*^2^) = 0.130	H-atom parameters constrained
*S* = 1.03	*w* = 1/[σ^2^(*F*_o_^2^) + (0.0771*P*)^2^ + 0.4377*P*] where *P* = (*F*_o_^2^ + 2*F*_c_^2^)/3
2933 reflections	(Δ/σ)_max_ < 0.001
206 parameters	Δρ_max_ = 0.29 e Å^−^^3^
30 restraints	Δρ_min_ = −0.34 e Å^−^^3^

### Special details

**Table:**

Geometry. All e.s.d.'s (except the e.s.d. in the dihedral angle between two l.s. planes) are estimated using the full covariance matrix. The cell e.s.d.'s are taken into account individually in the estimation of e.s.d.'s in distances, angles and torsion angles; correlations between e.s.d.'s in cell parameters are only used when they are defined by crystal symmetry. An approximate (isotropic) treatment of cell e.s.d.'s is used for estimating e.s.d.'s involving l.s. planes.
Refinement. Refinement of *F*^2^ against ALL reflections. The weighted *R*-factor *wR* and goodness of fit *S* are based on *F*^2^, conventional *R*-factors *R* are based on *F*, with *F* set to zero for negative *F*^2^. The threshold expression of *F*^2^ > σ(*F*^2^) is used only for calculating *R*-factors(gt) *etc*. and is not relevant to the choice of reflections for refinement. *R*-factors based on *F*^2^ are statistically about twice as large as those based on *F*, and *R*- factors based on ALL data will be even larger.

### Fractional atomic coordinates and isotropic or equivalent isotropic displacement parameters (Å^2^)

**Table:**

	*x*	*y*	*z*	*U*_iso_*/*U*_eq_	Occ. (<1)
O1	−0.40903 (12)	0.07719 (11)	−0.05516 (6)	0.0301 (3)	
O2	−0.31528 (12)	0.04014 (11)	0.07904 (6)	0.0274 (3)	
H2	−0.4096	−0.0008	0.0723	0.099 (10)*	
C1	−0.30126 (16)	0.08804 (13)	0.01415 (8)	0.0198 (3)	
C2	−0.14885 (15)	0.15904 (13)	0.02749 (8)	0.0191 (3)	
C3	−0.07123 (16)	0.22058 (13)	0.10232 (8)	0.0192 (3)	
H3	−0.1154	0.2127	0.1419	0.029*	
C4	0.06989 (16)	0.29353 (13)	0.12086 (8)	0.0192 (3)	
C5	0.13789 (16)	0.30168 (13)	0.06178 (8)	0.0196 (3)	
C6	0.05971 (16)	0.23646 (14)	−0.01268 (8)	0.0230 (3)	
H6	0.1069	0.2400	−0.0512	0.034*	
C7	−0.08302 (16)	0.16713 (14)	−0.03275 (8)	0.0219 (3)	
C8	−0.15681 (19)	0.10224 (17)	−0.11538 (9)	0.0316 (4)	
H8A	−0.2521	0.1494	−0.1483	0.047*	
H8B	−0.1846	0.0128	−0.1088	0.047*	
H8C	−0.0807	0.1032	−0.1416	0.047*	
C9	0.29440 (16)	0.37676 (14)	0.07460 (8)	0.0231 (3)	
C10A	0.3364 (4)	0.4704 (4)	0.1476 (2)	0.0292 (7)	0.683 (4)
H10A	0.2582	0.5428	0.1321	0.044*	0.683 (4)
C11A	0.3210 (3)	0.3998 (3)	0.21971 (13)	0.0272 (7)	0.683 (4)
H11A	0.3627	0.4560	0.2675	0.041*	0.683 (4)
H11B	0.3858	0.3202	0.2310	0.041*	0.683 (4)
C15A	0.5059 (12)	0.5308 (12)	0.1732 (6)	0.0497 (14)	0.683 (4)
H15A	0.5265	0.5866	0.2198	0.075*	0.683 (4)
H15B	0.5123	0.5819	0.1288	0.075*	0.683 (4)
H15C	0.5847	0.4615	0.1867	0.075*	0.683 (4)
C10B	0.3736 (8)	0.4188 (8)	0.1699 (4)	0.0292 (15)	0.317 (4)
H10B	0.4204	0.3404	0.2026	0.044*	0.317 (4)
C11B	0.2488 (7)	0.4758 (5)	0.1983 (3)	0.0297 (14)	0.317 (4)
H11C	0.1851	0.5417	0.1600	0.045*	0.317 (4)
H11D	0.3005	0.5171	0.2513	0.045*	0.317 (4)
C15B	0.507 (3)	0.524 (3)	0.1870 (15)	0.0497 (14)	0.317 (4)
H15D	0.4578	0.6093	0.1706	0.075*	0.317 (4)
H15E	0.5725	0.5038	0.1568	0.075*	0.317 (4)
H15F	0.5720	0.5256	0.2443	0.075*	0.317 (4)
C14A	0.0479 (4)	0.4785 (3)	0.20837 (16)	0.0297 (6)	0.683 (4)
H14A	−0.0611	0.4526	0.1978	0.045*	0.683 (4)
H14B	0.0488	0.5422	0.1684	0.045*	0.683 (4)
H14C	0.0947	0.5166	0.2619	0.045*	0.683 (4)
C14B	−0.0085 (8)	0.4464 (7)	0.2128 (4)	0.0297 (6)	0.317 (4)
H14D	0.0298	0.4937	0.2635	0.045*	0.317 (4)
H14E	−0.0922	0.3865	0.2118	0.045*	0.317 (4)
H14F	−0.0504	0.5075	0.1683	0.045*	0.317 (4)
C12	0.14176 (18)	0.36242 (15)	0.20370 (8)	0.0261 (3)	
C13	0.1649 (2)	0.26665 (17)	0.27301 (9)	0.0393 (4)	
H13A	0.2261	0.3084	0.3241	0.059*	
H13B	0.2217	0.1902	0.2663	0.059*	
H13C	0.0617	0.2407	0.2725	0.059*	
C16	0.42374 (19)	0.27774 (17)	0.07668 (13)	0.0408 (4)	
H16A	0.5219	0.3237	0.0843	0.061*	
H16B	0.3887	0.2300	0.0261	0.061*	
H16C	0.4420	0.2172	0.1210	0.061*	
C17	0.2657 (2)	0.4705 (2)	0.00255 (13)	0.0492 (5)	
H17A	0.1800	0.5304	−0.0016	0.074*	
H17B	0.2367	0.4204	−0.0470	0.074*	
H17C	0.3623	0.5195	0.0111	0.074*	

### Atomic displacement parameters (Å^2^)

**Table:**

	*U*^11^	*U*^22^	*U*^33^	*U*^12^	*U*^13^	*U*^23^
O1	0.0218 (5)	0.0408 (6)	0.0227 (5)	−0.0104 (4)	0.0028 (4)	−0.0008 (4)
O2	0.0213 (5)	0.0348 (6)	0.0255 (5)	−0.0064 (4)	0.0081 (4)	0.0061 (4)
C1	0.0183 (6)	0.0202 (6)	0.0202 (6)	−0.0005 (5)	0.0066 (5)	0.0002 (5)
C2	0.0172 (6)	0.0191 (7)	0.0203 (6)	−0.0001 (5)	0.0063 (5)	0.0007 (5)
C3	0.0206 (6)	0.0198 (7)	0.0187 (6)	0.0008 (5)	0.0092 (5)	0.0012 (5)
C4	0.0200 (6)	0.0170 (6)	0.0185 (6)	−0.0001 (5)	0.0050 (5)	−0.0002 (5)
C5	0.0177 (6)	0.0175 (6)	0.0227 (6)	−0.0001 (5)	0.0067 (5)	0.0012 (5)
C6	0.0238 (7)	0.0266 (7)	0.0223 (7)	−0.0025 (5)	0.0130 (6)	−0.0021 (5)
C7	0.0228 (7)	0.0233 (7)	0.0197 (6)	−0.0024 (5)	0.0081 (5)	−0.0024 (5)
C8	0.0328 (8)	0.0414 (9)	0.0230 (7)	−0.0119 (7)	0.0135 (6)	−0.0100 (6)
C9	0.0188 (7)	0.0235 (7)	0.0271 (7)	−0.0041 (5)	0.0088 (5)	−0.0006 (6)
C10A	0.0318 (16)	0.0283 (16)	0.0270 (15)	−0.0133 (12)	0.0106 (12)	−0.0039 (12)
C11A	0.0238 (12)	0.0336 (15)	0.0201 (10)	−0.0111 (11)	0.0038 (9)	−0.0039 (10)
C15A	0.0487 (12)	0.068 (2)	0.036 (4)	−0.0383 (13)	0.019 (2)	−0.016 (2)
C10B	0.026 (3)	0.026 (3)	0.028 (3)	−0.006 (2)	0.001 (2)	0.004 (2)
C11B	0.038 (3)	0.023 (3)	0.024 (2)	−0.006 (2)	0.006 (2)	0.000 (2)
C15B	0.0487 (12)	0.068 (2)	0.036 (4)	−0.0383 (13)	0.019 (2)	−0.016 (2)
C14A	0.0400 (18)	0.0209 (14)	0.0221 (9)	−0.0016 (11)	0.0048 (11)	−0.0056 (9)
C14B	0.0400 (18)	0.0209 (14)	0.0221 (9)	−0.0016 (11)	0.0048 (11)	−0.0056 (9)
C12	0.0300 (7)	0.0291 (8)	0.0189 (6)	−0.0107 (6)	0.0087 (5)	−0.0050 (6)
C13	0.0526 (10)	0.0349 (9)	0.0199 (7)	0.0117 (8)	0.0020 (7)	0.0004 (6)
C16	0.0247 (8)	0.0343 (9)	0.0677 (12)	−0.0011 (7)	0.0224 (8)	0.0013 (8)
C17	0.0290 (9)	0.0488 (11)	0.0625 (12)	−0.0103 (8)	0.0092 (8)	0.0271 (9)

### Geometric parameters (Å, °)

**Table:**

O1—C1	1.2458 (17)	C15A—H15B	0.9600
O2—C1	1.2969 (16)	C15A—H15C	0.9600
O2—H2	0.9088	C10B—C11B	1.505 (9)
C1—C2	1.4830 (18)	C10B—C15B	1.546 (12)
C2—C3	1.3892 (18)	C10B—H10B	0.9800
C2—C7	1.4040 (18)	C11B—C12	1.524 (5)
C3—C4	1.3939 (19)	C11B—H11C	0.9700
C3—H3	0.9300	C11B—H11D	0.9700
C4—C5	1.3992 (19)	C15B—H15D	0.9600
C4—C12	1.5289 (18)	C15B—H15E	0.9600
C5—C6	1.4020 (19)	C15B—H15F	0.9600
C5—C9	1.5366 (18)	C14A—C12	1.468 (3)
C6—C7	1.3839 (19)	C14A—H14A	0.9600
C6—H6	0.9300	C14A—H14B	0.9600
C7—C8	1.5089 (18)	C14A—H14C	0.9600
C8—H8A	0.9600	C14B—C12	1.653 (7)
C8—H8B	0.9600	C14B—H14D	0.9600
C8—H8C	0.9600	C14B—H14E	0.9600
C9—C16	1.525 (2)	C14B—H14F	0.9600
C9—C10A	1.532 (3)	C12—C13	1.516 (2)
C9—C17	1.533 (2)	C13—H13A	0.9600
C9—C10B	1.620 (6)	C13—H13B	0.9600
C10A—C11A	1.515 (4)	C13—H13C	0.9600
C10A—C15A	1.540 (7)	C16—H16A	0.9600
C10A—H10A	0.9800	C16—H16B	0.9600
C11A—C12	1.570 (3)	C16—H16C	0.9600
C11A—H11A	0.9700	C17—H17A	0.9600
C11A—H11B	0.9700	C17—H17B	0.9600
C15A—H15A	0.9600	C17—H17C	0.9600
			
C1—O2—H2	117.0	C15B—C10B—C9	112.8 (11)
O1—C1—O2	122.65 (12)	C11B—C10B—H10B	108.7
O1—C1—C2	121.59 (12)	C15B—C10B—H10B	108.7
O2—C1—C2	115.76 (11)	C9—C10B—H10B	108.7
C3—C2—C7	119.68 (12)	C10B—C11B—C12	107.3 (4)
C3—C2—C1	117.92 (11)	C10B—C11B—H11C	110.3
C7—C2—C1	122.39 (12)	C12—C11B—H11C	110.3
C2—C3—C4	123.01 (12)	C10B—C11B—H11D	110.3
C2—C3—H3	118.5	C12—C11B—H11D	110.3
C4—C3—H3	118.5	H11C—C11B—H11D	108.5
C3—C4—C5	118.05 (12)	C10B—C15B—H15D	109.5
C3—C4—C12	118.89 (12)	C10B—C15B—H15E	109.5
C5—C4—C12	123.05 (12)	H15D—C15B—H15E	109.5
C4—C5—C6	118.07 (12)	C10B—C15B—H15F	109.5
C4—C5—C9	123.51 (12)	H15D—C15B—H15F	109.5
C6—C5—C9	118.41 (11)	H15E—C15B—H15F	109.5
C7—C6—C5	124.41 (12)	C12—C14A—H14A	109.5
C7—C6—H6	117.8	C12—C14A—H14B	109.5
C5—C6—H6	117.8	C12—C14A—H14C	109.5
C6—C7—C2	116.73 (12)	C12—C14B—H14D	109.5
C6—C7—C8	119.56 (12)	C12—C14B—H14E	109.5
C2—C7—C8	123.70 (12)	C12—C14B—H14F	109.5
C7—C8—H8A	109.5	C14A—C12—C13	111.94 (16)
C7—C8—H8B	109.5	C13—C12—C11B	129.6 (2)
H8A—C8—H8B	109.5	C14A—C12—C4	112.25 (15)
C7—C8—H8C	109.5	C13—C12—C4	111.21 (12)
H8A—C8—H8C	109.5	C11B—C12—C4	109.3 (2)
H8B—C8—H8C	109.5	C14A—C12—C11A	111.49 (18)
C16—C9—C10A	116.56 (19)	C13—C12—C11A	101.08 (15)
C16—C9—C17	108.36 (14)	C4—C12—C11A	108.28 (12)
C10A—C9—C17	103.15 (19)	C13—C12—C14B	96.8 (3)
C16—C9—C5	108.75 (12)	C11B—C12—C14B	100.0 (3)
C10A—C9—C5	110.50 (14)	C4—C12—C14B	105.4 (2)
C17—C9—C5	109.21 (12)	C12—C13—H13A	109.5
C16—C9—C10B	96.9 (3)	C12—C13—H13B	109.5
C17—C9—C10B	124.9 (3)	H13A—C13—H13B	109.5
C5—C9—C10B	107.4 (2)	C12—C13—H13C	109.5
C11A—C10A—C9	110.2 (2)	H13A—C13—H13C	109.5
C11A—C10A—C15A	109.7 (4)	H13B—C13—H13C	109.5
C9—C10A—C15A	113.2 (5)	C9—C16—H16A	109.5
C11A—C10A—H10A	107.9	C9—C16—H16B	109.5
C9—C10A—H10A	107.9	H16A—C16—H16B	109.5
C15A—C10A—H10A	107.9	C9—C16—H16C	109.5
C10A—C11A—C12	112.1 (2)	H16A—C16—H16C	109.5
C10A—C11A—H11A	109.2	H16B—C16—H16C	109.5
C12—C11A—H11A	109.2	C9—C17—H17A	109.5
C10A—C11A—H11B	109.2	C9—C17—H17B	109.5
C12—C11A—H11B	109.2	H17A—C17—H17B	109.5
H11A—C11A—H11B	107.9	C9—C17—H17C	109.5
C11B—C10B—C15B	106.6 (14)	H17A—C17—H17C	109.5
C11B—C10B—C9	111.2 (5)	H17B—C17—H17C	109.5
			
O1—C1—C2—C3	150.42 (13)	C5—C9—C10A—C15A	170.1 (5)
O2—C1—C2—C3	−28.69 (18)	C10B—C9—C10A—C15A	83.1 (9)
O1—C1—C2—C7	−28.8 (2)	C9—C10A—C11A—C12	−67.0 (3)
O2—C1—C2—C7	152.14 (13)	C15A—C10A—C11A—C12	167.7 (6)
C7—C2—C3—C4	1.2 (2)	C16—C9—C10B—C11B	−157.8 (5)
C1—C2—C3—C4	−177.96 (12)	C10A—C9—C10B—C11B	55.8 (7)
C2—C3—C4—C5	−1.7 (2)	C17—C9—C10B—C11B	84.1 (5)
C2—C3—C4—C12	177.34 (12)	C5—C9—C10B—C11B	−45.6 (6)
C3—C4—C5—C6	0.27 (19)	C16—C9—C10B—C15B	82.6 (15)
C12—C4—C5—C6	−178.78 (13)	C10A—C9—C10B—C15B	−63.9 (16)
C3—C4—C5—C9	−178.97 (12)	C17—C9—C10B—C15B	−35.5 (15)
C12—C4—C5—C9	2.0 (2)	C5—C9—C10B—C15B	−165.3 (14)
C4—C5—C6—C7	1.8 (2)	C15B—C10B—C11B—C12	−164.9 (11)
C9—C5—C6—C7	−178.93 (13)	C9—C10B—C11B—C12	71.8 (6)
C5—C6—C7—C2	−2.3 (2)	C10B—C11B—C12—C14A	−166.0 (4)
C5—C6—C7—C8	179.27 (14)	C10B—C11B—C12—C13	85.6 (4)
C3—C2—C7—C6	0.74 (19)	C10B—C11B—C12—C4	−56.4 (5)
C1—C2—C7—C6	179.90 (12)	C10B—C11B—C12—C11A	38.6 (4)
C3—C2—C7—C8	179.11 (13)	C10B—C11B—C12—C14B	−166.8 (5)
C1—C2—C7—C8	−1.7 (2)	C3—C4—C12—C14A	−73.3 (2)
C4—C5—C9—C16	113.07 (15)	C5—C4—C12—C14A	105.7 (2)
C6—C5—C9—C16	−66.16 (17)	C3—C4—C12—C13	52.99 (17)
C4—C5—C9—C10A	−16.1 (2)	C5—C4—C12—C13	−127.97 (15)
C6—C5—C9—C10A	164.7 (2)	C3—C4—C12—C11B	−157.5 (3)
C4—C5—C9—C17	−128.86 (16)	C5—C4—C12—C11B	21.5 (3)
C6—C5—C9—C17	51.91 (18)	C3—C4—C12—C11A	163.19 (15)
C4—C5—C9—C10B	9.2 (3)	C5—C4—C12—C11A	−17.8 (2)
C6—C5—C9—C10B	−170.0 (3)	C3—C4—C12—C14B	−50.8 (3)
C16—C9—C10A—C11A	−78.0 (3)	C5—C4—C12—C14B	128.2 (3)
C17—C9—C10A—C11A	163.4 (2)	C10A—C11A—C12—C14A	−74.4 (3)
C5—C9—C10A—C11A	46.8 (3)	C10A—C11A—C12—C13	166.5 (2)
C10B—C9—C10A—C11A	−40.1 (6)	C10A—C11A—C12—C11B	−48.5 (3)
C16—C9—C10A—C15A	45.3 (6)	C10A—C11A—C12—C4	49.6 (3)
C17—C9—C10A—C15A	−73.3 (6)	C10A—C11A—C12—C14B	−83.4 (4)

### Hydrogen-bond geometry (Å, °)

**Table:**

*D*—H···*A*	*D*—H	H···*A*	*D*···*A*	*D*—H···*A*
O2—H2···O1^i^	0.91	1.72	2.6305 (16)	178

Symmetry codes: (i) −*x*−1, −*y*, −*z*.
